# Healthcare reform: implications for knowledge translation in primary care

**DOI:** 10.1186/1472-6963-13-490

**Published:** 2013-11-25

**Authors:** Ann Dadich, Hassan Hosseinzadeh

**Affiliations:** 1 School of Business, University of Western Sydney, Locked Bag 1797, Parramatta, NSW, Australia 2751

**Keywords:** Knowledge translation, Healthcare reform, Evidence-based practice, Primary care, Sexual healthcare

## Abstract

**Background:**

The primary care sector represents the linchpin of many health systems. However, the translation of evidence-based practices into patient care can be difficult, particularly during healthcare reform. This can have significant implications for patients, their communities, and the public purse. This is aptly demonstrated in the area of sexual health. The aim of this paper is to determine what works to facilitate evidence-based sexual healthcare within the primary care sector.

**Methods:**

431 clinicians (214 general practitioners and 217 practice nurses) in New South Wales, Australia, were surveyed about their awareness, their use, the perceived impact, and the factors that hindered the use of six resources to promote sexual healthcare. Descriptive statistics were calculated from the responses to the closed survey items, while responses to open-ended item were thematically analyzed.

**Results:**

All six resources were reported to improve the delivery of evidence-based sexual healthcare. Two resources – both double-sided A4-placards – had the greatest reach and use. Barriers that hindered resource-use included limited time, limited perceived need, and limited access to, or familiarity with the resources. Furthermore, the reorganization of the primary care sector and the removal of particular medical benefits scheme items may have hampered clinician capacity to translate evidence-based practices into patient care.

**Conclusions:**

Findings reveal: (1) the translation of evidence-based practices into patient care is viable despite reform; (2) the potential value of a multi-modal approach; (3) the dissemination of relatively inexpensive resources might influence clinical practices; and (4) reforms to governance and/or funding arrangements may widen the void between evidence-based practices and patient care.

## Background

Internationally, health systems with a stronger primary care sector are relatively more effective and efficient, and generate better patient outcomes [1]. This is particularly important given contemporary challenges – like ‘increased rates of chronic and preventable disease, new treatments becoming available and rising health care costs’ [2], para. 1.

For these (and perhaps other) reasons, many Western governments have endeavored to reform, and ultimately strengthen their nation’s primary care sector [3-7]. They have vied for ways to improve the organization, management, and delivery of healthcare [8]. For instance, Australia is currently witnessing ‘the single biggest health reform in a quarter of a century’ [9], p. 2, the essence of which is healthcare that is ‘funded nationally and run locally’ [10], p. 25. Towards this aim, the national government is working with state and territory governments to reinforce the primary care sector as the linchpin of the health system [11,12]. This is epitomized by the establishment of Medicare Locals – independent primary care organizations with a mandate to provide locally-responsive, planned, and coordinated primary care services. Since 2011, 61 Medicare Locals have been established across Australia, all of which aim to: improve the patient journey by developing integrated and coordinated services; support clinicians and other practitioners to improve patient care; address local health needs; ensure the effective implementation of primary care initiatives; as well as ensure efficiency and accountability [13]. This broader remit of the primary care sector – whereby health promotion, prevention, and early intervention are provided in tandem with treatment and disease management – is expected reduce Australia’s hospital-centric health system.

Prior to these current healthcare reforms, Australia witnessed many others [14-19]. The nation’s relatively short and recent history includes the establishment of the Hospitals and Health Services Commission and the Health Insurance Commission in the 1970s, which were accompanied by the introduction of Medibank – the government-owned private health insurer. This history also includes the launch of Medicare in the 1980s – a universal health insurance scheme to make healthcare affordable for all Australians. Following the turn of the century, Medical Indemnity Acts were introduced to curb the rise in negligence and malpractice claims – furthermore, Medicare Australia was formed to increase access to multidisciplinary health services coordinated by general practitioners (GPs), particularly for people with complex or chronic conditions. Given its rate of recurrence, it might be argued that healthcare reform is now routine [20].

As an exercise in change – euphemisms for which include reorganization, rationalization, and restructuring [20] – healthcare reform is likely to be associated with volatility and instability [21,22]. This includes uncertainty [23-25], diminished morale [26,27], and staff turnover [28]. Such an environment may distract healthcare organizations from their core business [29-31] including the delivery of quality healthcare through the use of evidence-based practices.

Optimizing clinician use of evidence-based practice represents a significant challenge within healthcare services [32] – this includes primary care. One of the key issues within primary care is to effectively and efficiently translate evidence from empirical research into patient care [33-36]. Although research focused solely on primary care is limited, research suggests that clinician use of evidence-based practice is problematic [37,38]. For instance, a recent Australian study concluded that the delivery of evidence-based care is less than ideal. The authors stated, ‘Compliance with indicators of appropriate care at condition level ranged from 13%… for alcohol dependence to 90%… for coronary artery disease… Although there were pockets of excellence… the consistent delivery of appropriate care needs improvement’ [39], p. 100.

There are a myriad of reasons that contribute to this ‘quality chasm’ [40-42] – these include doctor-related, patient-related, and organizational factors. For instance, in Australia following a cluster randomized controlled trial on chlamydia screening in general practice, Bowden and colleagues [43] concluded that limited time, limited clinician understanding of associated benefits, and clinician concern about broaching sexual health with patients hindered clinician capacity to deliver evidence-based sexual healthcare.

The limited use of evidence-based practice has significant consequences for patients, their communities, and the public purse [41]. This is largely because evidence-based practice is said to enhance quality patient care (at least at the individual level) and optimize the allocation of limited resources [44-46]. This might partly explain current government and academic interest in knowledge translation [47].

Despite the myriad of terms coined to refer to knowledge translation – including research utilization, implementation, dissemination, and diffusion, among others [48] – the term might be understood as ‘any activity or process that facilitates the transfer of high-quality evidence from research into effective changes in health policy, clinical practice, or products’ [49]. Although the ultimate aim of knowledge translation is to use (near) irrefutable evidence to improve patient care, this translation (*translation* being the operative word) is a complex, dynamic, and an evolving process [50]. To facilitate this process effectively and efficiently, international scholars have called for broad approaches [47] and innovative methods [51], lessons for which might be garnered from extant research. For instance, a comprehensive review of extant literature suggests that most methods to help clinicians and practitioners to adopt evidence-based practices have the capacity to effect change – however, robust evidence of their effectiveness (and methods of action) is lacking [45]. Although the evidence for effective methods remains inconclusive, it does not suggest that particular methods be discontinued [52]. Rather, there are ‘no “magic bullets” for improving the quality of health care’ [53], p. 1423. Bridging the divide between evidence-based practice and patient care appears to require a multimodal approach. As Grol and Grimshaw concluded, ‘Different types of changes seem to need discrete types of interventions… research so far shows that none of the approaches is superior for all changes in all situations; we probably need them all’ [54], pp. 1227–1229. Therefore, different methods are likely to be required for different audiences, for different purposes, and at different times – this includes times of significant organizational change.

To better understand what works when facilitating knowledge translation – particularly during a time of considerable healthcare reform [55] – this study presents findings from a recent survey of GPs and practice nurses (PNs) in Australia about their awareness, their use, the perceived impact, and the factors that hindered the use of six resources to promote sexual healthcare. Sexual healthcare in the Australian primary care sector constitutes an appropriate context for three key reasons. First, despite the prevalence of sexually transmissible infections [56-59], the delivery of sexual healthcare is limited, particularly within primary care [60-63]. This can have serious implications as some STIs remain asymptomatic and have long-term effects if left untreated [64,65]. Second, the Australian primary care sector is experiencing significant reform, the aim of which is to ‘shift the centre of gravity of the health system from hospitals to primary health care’ [66], p. 1. Third, primary care clinicians are being called to alleviate the strain on public sexual health clinics [67]. As stated in a government sexual health strategy, ‘The size of some priority population groups is such that a strategic objective for specialist clinics and Area-based sexual health programs must be to work with general practice to reduce barriers to access’ [68], p. 2. These three reasons lend sexual healthcare in the Australian primary care sector as an appropriate context for this study.

### GP Project

The New South Wales (NSW) Sexually Transmissible Infections Programs Unit (STIPU) developed and deployed the GP Project (in collaboration with key stakeholders) to enhance evidence-based sexual healthcare within general practice in NSW. Its objectives were to increase clinician access to STI information, education, and resources; promote their understanding of contact tracing; and clarify referral pathways. To meet these objectives, seven resources were developed for GPs and two for PNs, all of which were informed by clinical guidelines [69]. Given their similarities as educational aides (particularly in content), this paper reports on findings pertaining to six of these resources – namely, the STI Testing Tool, the Online STI Testing Tool GP Training, the Active Learning Module, the Check Booklet, the Practice Nurse Postcard, and the Online STI Practice Nurse Training.

**Figure 1:**
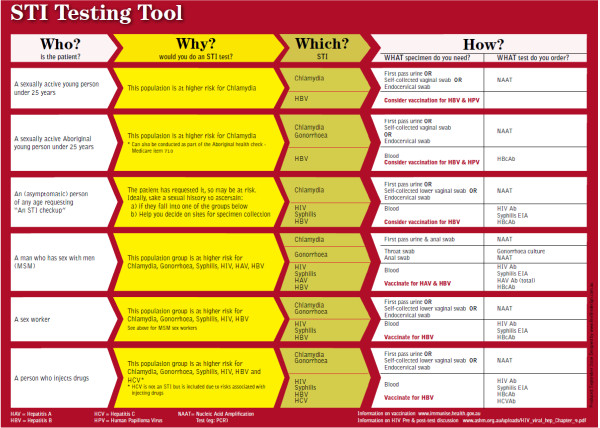
STI testing tool.

The *STI Testing Tool* is a double-sided A4 placard that guides sexual health consultations (see Figure 1). This includes the identification of at-risk patients; appropriate screening tests and the specimens required; appropriate ways to initiate and manage a sexual health consultation; a guide to documenting a brief sexual history; appropriate ways to broach contact tracing; as well as referral information. Following its development, the STI Testing Tool was promoted and disseminated via key professional bodies that support NSW GPs and promote general practice, and distributed to NSW GPs.

**Figure 2:**
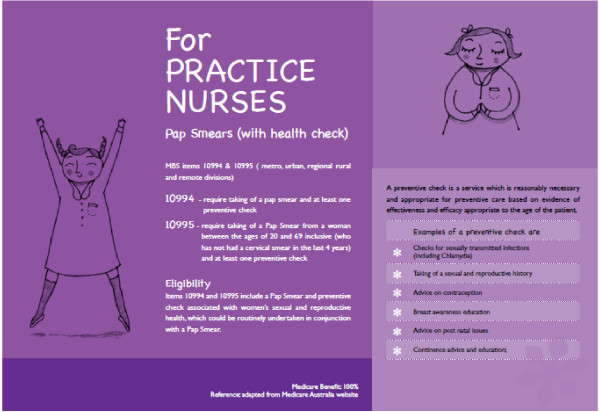
Practice nurse postcard.

Developed and distributed by an independent provider of online education to healthcare providers, the *Online STI Testing Tool GP Training* is an interactive course, which takes approximately sixty minutes to complete (see Figure 2). It includes seven clinical cases offering participants an opportunity to apply their skills and knowledge; these abilities are tested through the completion of questions after each clinical case, answers for which are also provided. Following its development, the Online STI Testing Tool GP Training was promoted electronically to GPs via website postings and email. It was delivered online by the independent provider as part of its training program [70].

The *Active Learning Module* is a face-to-face program comprised of three interactive educational modules to improve knowledge of, and clinical skills in STI management. Each two-hour module has a particular focus and builds on the preceding module. Although participants are awarded four continuing professional development (CPD) points for completing one module, forty CPD points are awarded following the completion of all three modules. The Active Learning Module was designed to foster interagency collaboration – more specifically, it aimed to encourage the 33 NSW Divisions of General Practice to work with the Australasian Society of HIV Medicine (ASHM), which delivered the modules. Divisions of General Practice are professional bodies that support members through the provision of training, resources, and opportunities to collaborate with other health professionals [71]. Following its development, the Active Learning Module was promoted via key stakeholders, including the Divisions, and at time of study, it was delivered on six occasions.

The *Check Booklet* on STIs was developed in accordance with the Royal Australian College of General Practitioners Quality Improvement and Continuing Professional Development program [72]. Check is an independent learning program published monthly by the RACGP on different health topics. This particular issue addresses: documenting a sexual history; STI testing; dealing with feelings of discomfort around sex; STI screening; contact tracing; and cultural sensitivities. The booklet includes seven clinical cases accompanied by questions and answers, and concludes with additional GP resources. As part of the RACGP Check program [73], the Check Booklet was promoted and delivered by the RACGP to GP members as part of their membership package.

The *Practice Nurse Postcard* was designed to help PNs undertake a preventative women’s health check, including a pap smear. Akin to the STI Testing Tool, it is designed as a double-sided A4 placard, which provides information on the health check, including medical benefits scheme (MBS) item numbers; prompts to document a brief sexual history; information to support the management of chlamydia with information on priority populations, screening tests, treatment, and prevention; and contact details for further resources. The postcard was promoted by relevant professional bodies, including the Australian Practice Nurses Association (APNA), and was disseminated as a paper-based postcard to general practices and clinicians. To expedite distribution, particularly to rural and remote areas, the postcard was also available online.

As part of the APNA Online Training program [74], the *Online STI Practice Nurse Training* is an interactive course that focuses on understanding and managing STIs, blood borne viruses (BBVs), human immunodeficiency virus (HIV), as well as viral hepatitis. This information is conveyed and reinforced via text, illustrations, graphs, charts, clinical cases, and hyperlinks to additional resources, including academic journal articles and websites. The training was promoted by relevant professional bodies, including ASHM, and was delivered by APNA in an online environment.

Following the development of these six resources, this study was conducted to determine GP and PN awareness, use, perceived impact on clinical practices, as well as factors that hindered use.

## Methods

Following clearance from the University of Western Sydney Human Research Ethics Committee (approval number: H8886), GPs and PNs practicing in NSW were recruited with the assistance of relevant professional bodies, as well as an independent provider of online education. These organizations included project information in its communications to GPs and PNs, which included email, facsimiles, website postings, and newsletters.

GPs and PNs were invited to complete an anonymous and a confidential online survey, comprised of closed and open-ended items. In addition to demographic information (about the respondent and their primary workplace), survey items pertained to resources within the GP Project. The purpose of the survey was to determine the degree of awareness; use of the resources; perceived impact on clinical practice; perceived value of the resources; perceived capacity to promote sexual health; and preferred learning styles. In recognition of their contribution to this project, respondents were offered hard copies of clinical guidelines. Data were collected for five months (August 2011 to January 2012).

Data collected through the closed survey items were cleaned. Descriptive statistics were then calculated using only valid responses – this includes the calculation of percentages and means. Akin to first-level coding [75], the second author initially reviewed the responses to each open-ended item to identify key elements and concepts; these were then discussed with the lead author and there were no discrepant views during this process. Both authors then distilled the elements and concepts into constructed themes, akin to axial coding [76]. Given the scope of this paper, only findings pertaining to awareness, use, perceived impact on clinical practices, and factors that hindered resource use are reported.

## Results

### Participants

A total of 431 primary care clinicians practicing in NSW completed the online survey – this includes 214 GPs and 217 PNs (see Table 1). Most respondents were female (GPs: 54.1%, PNs: 93.9%) and most graduated in Australia (GPs: 57.2%, PNs: 86.9%). The highest proportion of GPs was between 36 and 45 years of age (31.8%), and collectively they had an average of 15.4 years of GP experience (*SD* = 12.1). The highest proportion of PNs was between 41 and 50 years of age (35.5%), and together they had an average of seven years of PN experience (*SD* = 7.6). For most respondents, ten to fifty percent of their patients were under 25 years of age (GPs: 77.5%, PNs: 59.5%), and few of their patients were Indigenous (GPs: <1% = 54.5%; PNs: <5% = 74.6%). This is noteworthy given that sexually active young people – particularly Indigenous young people – represent a cohort at-risk of STIs [69].

**Table 1:** **Respondent socio-demographic characteristics (*n*** **= 431; GPs = 214; PNs = 217)**

**GP characteristics**	**%**	**PN characteristics**	**%**
Sex		Sex	
Male	45.9	Male	6.1
Female	54.1	Female	93.9
Age (yrs)		Age (yrs)	
26-35	15.4	20-30	12.4
36-45	31.8	31-40	18.5
46-55	29.4	41-50	35.5
>56	23.4	51-60	28.6
		>60	5.1
Country of graduation		Country of graduation	
Australia	57.2	Australia	86.9
Overseas	42.8	Overseas	13.1
Patients <25 yrs		Patients <25 yrs	
<10%	11.7	<10%	26.0
10-50%	77.5	10-50%	59.5
>50%	7.5	>50%	7.4
Unsure	3.3	Unsure	7.1
Indigenous patients		Indigenous patients	
<1%	54.5	<1%	44.1
1-5%	29.1	1-5%	30.5
5-20%	8.0	5-20%	8.0
>20%	5.2	>20%	6.6
Unsure	3.3	Unsure	10.8

Given the profile of NSW GPs [77], the demographic characteristics suggest the GP survey respondents were not representative of this cohort. This is because NSW GPs are mostly male (63.1%) and approximately one-third are over 55 years of age (31.6%). In the absence of detailed demographic data on the profile of NSW PNs [77], it is difficult to ascertain whether the PN survey respondents were representative of this cohort. However, data on the gender and age of all NSW registered nurses would suggest the survey respondents were not entirely representative of this cohort. This is because, although NSW registered nurses are mostly female (89.6%) (akin to the PN survey respondents), approximately one-fifth are over 55 years of age (21.2%), which differs from the PN survey respondents. Although the GP and PN survey respondents do not constitute a representative sample of Australian GPs or PNs, they supported diverse patient populations, including young people and Indigenous people.

### STI testing tool

Most GP respondents were aware of the STI Testing Tool (61.7%, see Table 2) and a majority of them used it (71.7%). Of those who used it, most indicated that it assisted their clinical practice (85.6%); improved their ability to raise the topic of STIs with patients (68.5%); and/or enhanced their ability to order appropriate STI tests (80.0%). According to respondents who were aware of, but did not use this resource, this was largely due to a perceived lack of need, limited access to the resource, time constraints, and limited familiarity with the resource. Respondents who used this resource indicated their ability to use it was hindered by limited access; they could not readily locate the resource when required, and/or they failed to remember its availability. These (and other) factors were exacerbated by their large workloads, which afforded them limited time.

**Table 2:** Awareness, use and perceived impact of resources (n = 431; GPs = 214; PNs = 217)

	**STI testing tool (%)**	**Online STI testing tool GP training (%)**	**Active learning module (%)**	**Check booklet (%)**	**Practice nurse postcard (%)**	**Online STI practice nurse training (%)**
Aware of resource	61.7	23.4	12.4	50.5	38.2	50.2
Used resource	71.7	28.3	29.2	53.5	63.5	18.7
Assisted clinical practice	85.6	83.3	100.0	86.8	63.0	82.4
Improved ability to raise sexual healthcare/contact tracing with patients	68.5	81.8	100.0			
Improved ability to identity/order appropriate STI tests	80.0	83.3	83.3			
Improved knowledge				90.2		
Improved ability to document brief sexual history			83.3	88.5		
Improved ability to identify at-risk patients			83.3			81.3
Improved ability to diagnose/treat common STIs			85.7			
Improved ability to undertake and claim for pap smears and preventative checks					63.8	
Improved ability to identify who should be tested for chlamydia					76.6	
Improved ability to test for chlamydia					68.9	
Improved ability to document brief sexual history					72.3	87.5
Improved knowledge of chlamydia treatment and prevention					67.4	
Improved ability to perform contact tracing						62.5
Improved ability to consult patients about testing, treating and preventing STIs						70.6

### Online STI testing tool GP training

Less than one-quarter of the GP respondents were aware of the Online STI Testing Tool GP Training (23.4%, see Table 2) and of these, less than one-third used it (28.3%). Most respondents who used this resource reported that it aided their clinical practice (83.3%); improved their ability to raise the topic of STIs with patients (81.8%); and/or enhanced their ability to order appropriate STI tests (83.3%). According to respondents who were aware of, but did not use this resource, key barriers were time constraints, workload, the increasing number of online training opportunities, and limited internet access, particularly in rural areas.

### Active learning module

Just over ten percent of GP respondents were aware of the Active Learning Module (12.4%, see Table 2); of these, less than one-third completed all three modules (29.2%). All of those who completed the three modules indicated the resource aided their clinical practice (100.0%). Most of these respondents suggested it improved their ability to document a brief sexual history (83.3%); identify patients at-risk of STIs (83.3%); identify appropriate STI tests (83.3%); diagnose and treat common STIs (85.7%); and raise contact tracing with patients (100.0%). According to respondents who were aware of, but did not use this resource, time constraints were the key barrier.

### Check booklet

Approximately one-half of the GP respondents were aware of the Check Booklet (50.5%) and more than half of these respondents read or completed it (53.5%). Most of the respondents who read or completed the booklet agreed that it aided their clinical practice (86.8%); improved their ability to document a brief sexual history (88.5%); improved their ability to diagnose and manage STIs (90.2%); and improved their understanding of cultural sensitivities when discussing STIs (83.0%). According to respondents who were aware of, but did not use the booklet, time constraints were said to be the key barrier.

### Practice nurse postcard

Over one-third of PN respondents were aware of the Practice Nurse Postcard (38.2%, see Table 2). Of these, close to two-thirds used it (63.5%). Most respondents who used the postcard agreed that it helped clinical practice (63.0%). Furthermore, most indicated that their knowledge of chlamydia treatment and prevention had improved (67.4%), as did their ability to undertake and claim for pap smears and preventative checks (63.8%); identify patients who should be tested for chlamydia (76.6%); test for chlamydia (68.9%); and document a brief sexual history (72.3%). According to respondents who were aware of, but did not use the postcard, limited access and limited relevance to their current role were key barriers. Similarly, respondents who used this item indicated that limited access and unfamiliarity with the ordering process hindered their ability to use it.

### Online STI practice nurse training

Approximately half of the PN respondents were aware of the Online STI Practice Nurse Training (50.2%, see Table 2) and less than one-fifth of these PNs completed it (18.7%). Most respondents who completed this training agreed that it helped clinical practice (82.4%). Most cited improvement in their capacity to document a brief sexual history (87.5%); identify patients at-risk of STIs (81.3%); perform contact tracing (62.5%); as well as consult patients about STI testing, treatment, and prevention (70.6%). Those who completed the item indicated cost and time constraints were the key barriers that hindered their ability to use the information during clinical practice.

## Discussion

In this epoch of primary care reform [27,78,79], which can distract from core clinical business [29-31], it is important to identify strategies that facilitate knowledge translation. Enabling clinicians to access timely, comprehensible information on evidence-based practice is likely to optimize its use, its influence on clinical decision-making and, as such, patient care [80-82]. This is because evidence-based practices – like clinical guidelines – meld clinical expertise with evidence borne from empirical research [83].

This paper presents findings from a recent study to examine the capacity of six resources to facilitate knowledge translation in primary care. GP resources included the STI Testing Tool, the Online STI Testing Tool GP Training, the Active Learning Module, and the Check Booklet, while PN resources included the Practice Nurse Postcard and the Online STI Practice Nurse Training. A survey of 431 GPs and PNs revealed three key findings. First, the highest proportions used their respective double-sided A4 placards – namely, the STI Testing Tool and the Practice Nurse Postcard – this may be due in part to their wide circulation, which included direct distribution to clinicians. Second, all six resources were perceived to assist clinical practice. Third, all six resources were perceived to improve clinical ability to deliver sexual healthcare – this includes the ability to broach sexual healthcare or contact tracing with patients; identity and/or order appropriate STI tests; document a brief sexual history; identify at-risk patients; as well as diagnose and/or treat common STIs.

The respondents reported several barriers that hindered their capacity to use the resources. Reflecting extant primary care research [84-88], these include limited time – largely due to workload and competing professional development priorities; limited perceived need – particularly among PNs who suggested there was limited relevance to their current role; as well as limited access to, or familiarity with the resource.

Further to these, recent reforms within the Australian primary care sector may have contributed to the ‘quality chasm’ [42]. During the course of the GP Project, two key changes occurred that has a direct bearing on the resources. The first was the transition of NSW Divisions of General Practice to Medicare Locals. As indicated, the Divisions played a key role in the promotion and delivery of the resources, serving as the conduit to GPs and PNs. By July 1, 2012, twenty Medicare Locals were established in NSW, replacing the Divisions with organizations charged with greater responsibility. Like the Divisions, Medicare Locals support members through the provision of training, resources, and opportunities to collaborate with other health professionals – however, their responsibilities also include ‘local health planning, identifying gaps in services at the local level, examining opportunities for better targeting of services and establishing formal and informal linkages with the acute and aged care sectors’ [89], p. 4. Although Medicare Locals ‘retain, and expand, the functions and activities… [of] the Divisions’, it would perhaps be naïve to assume this period of transition did not influence their capacity to actively engage with and promote the GP Project. This is largely because the transition involved a detailed application process, in which Divisions were required to demonstrate their expertise, capacity, and financial viability, as well as propose governance and operational arrangements and a strategic plan. In the face of an uncertain outcome, extant literature would suggest negative effects on staff morale [26,27] and staff turnover [28], which may in turn have diminished the potential of the GP Project. This is likely to have been the case for the Active Learning Module, which required collaboration between Divisions and ASHM.

Another key change was the removal of all MBS PN items pertaining to pap smears. On December 31, 2011, government reforms saw changes to ways in which organizations that employ a PN are funded. These included the cessation of MBS items that paid PNs for initiating and conducting a pap smear (items 10994, 10995, 10998, and 10999). Although PNs may still conduct a pap smear as part of a comprehensive health check for particular patient groups (as is the case with the Aboriginal Health Check), they are no longer funded for ‘task-oriented’ [90] , para. 8 services. This has direct implications for the Practice Nurse Postcard, which notes the (now redundant) MBS items. Although the remaining information may still be of value to PNs, it will only be of value during comprehensive health checks.

The findings from this study are important for four key reasons. First, it suggests that the translation of information on evidence-based sexual healthcare into patient care is possible during ‘the single biggest health reform in a quarter of a century’ [9], p. 2. Despite an uncertain climate [91,92], and the potential distraction from quality patient care [93,94], respondents reported a perceived change in their clinical capacities following the use of the resources.

Second, the findings reinforce the potential value of a multi-modal approach to knowledge translation. In accordance with extant literature [45,52-54], primary care clinicians – like GPs and PNs – are likely to recognize value in different approaches that communicate evidence-based practices.

Third, the findings suggest that the wide dissemination of relatively inexpensive resources – like the STI Testing Tool and the Practice Nurse Postcard – might influence clinical practices. Although cost-benefit and/or cost-effectiveness analyses [95,96] were beyond the scope of this study, one might assume that the costs associated with the production and postage of a double-sided A4 placard are likely to be economical, relative to the costs associated with the development, maintenance, and delivery of online training or an Active Learning Module. It therefore appears that relatively inexpensive resources might be an effective and efficient way to facilitate knowledge translation.

Finally, despite the seeming value of the resources, the findings allude to the dark-side of healthcare reform. Although the design of this study does not permit the identification of causal relationships, changes to governance and/or funding arrangements (e.g., the introduction of Medicare Locals and the cessation of some MBS items) may widen the void between evidence-based practices and patient care, at least in the short-term. While further research is required to explore this, additional strategies may be needed to facilitate knowledge translation. For instance, following a systematic review, Flodgren and colleagues [97] concluded that ‘Financial incentives may be effective in changing healthcare professional practice’ [97], p. 2. Yet, research is required to determine the conditions that optimize such change.

Despite the potential value of these findings, three methodological limitations deserve consideration. First, the respondents do not constitute a representative sample of Australian GPs or PNs [77,98] – as such, it is unlikely that findings are generalizable within and beyond the NSW primary care sector. Second, as voluntary, self-reporting participants, it is possible that respondents had a particular interest in sexual healthcare and were largely *au fait* with evidence-based practices, relative to their peers – as such, the views here presented may be biased, particularly given the use of self-reported data. Third, the cross-sectional nature of this study – particularly the use of a survey, indicates that the respondents provided an incomplete snapshot of their views, which might alter over time.

## Conclusions

This study contributes to the growing research on knowledge translation in the primary care sector [99-101]. In addition to the four aforesaid practical implications, it also provides a platform for future research to: (1) identify the factors that help and hinder knowledge translation during considerable reform; and (2) determine the potential strength of their influence on clinical practices, patient wellbeing, and public health. Given the plethora of confounding variables that can influence knowledge translation [102], and the complexities associated with community-based research [103], such research may require a mixed-method design that draws from a blend of appropriate methodologies, including (but not limited to) cluster randomized controlled trials [104,105] that encompasses ethnography and/or participatory action research [106]. This might involve co-creating resources with clinicians and consumers and testing their effectiveness for the same cohort in two or more locales, distinguished primarily by the characteristics of the primary care service. This is not to suggest that this design is problem-free [107,108] – however, it represents one approach to better understand and ultimately improve evidence-based primary care during a time of significant reform.

## Competing interests

The authors declare they have no competing interests.

## Authors’ contributions

AD conceived, led, and supervised the study and this manuscript, particularly the Background, Methods, Discussion, and Conclusion sections. HH assisted with the collection and analysis of the data and developed the Results section. Both authors read and approved the final manuscript.

## Pre-publication history

The pre-publication history for this paper can be accessed here:

http://www.biomedcentral.com/1472-6963/13/490/prepub
